# Giant right atrial hemangioma presenting with ascites: A case report

**DOI:** 10.1515/med-2025-1169

**Published:** 2025-07-23

**Authors:** Antonio Salsano, Giacomo Perocchio, Antonio Guadagno, Paolo Nozza, Tommaso Regesta, Francesco Santini

**Affiliations:** Division of Cardiac Surgery, IRCCS Ospedale Policlinico San Martino, DISC Department, University of Genoa, 16132, Genoa, Italy; DISC Department, University of Genoa, 16126, Genoa, Italy; Pathology Unit, IRCCS Ospedale Policlinico San Martino, Genova, Italy

**Keywords:** hemangioma, cardiac turmour, ascites, right atrium

## Abstract

**Introduction:**

Cardiac hemangiomas are slow-growing benign tumours of the heart. Patients may be asymptomatic or present a multitude of signs or symptoms.

**Methods:**

We report herein the case of a 72-year-old woman with a giant right atrial mass. The patient suffers from abdominal swelling related to ascites. The histological examination of the tranjugular biopsy suspected an atrial myxoma.

**Results:**

The patient was scheduled for surgical excision of the cardiac tumour. Radical resection of a 13 cm mass was performed. The histological diagnosis revealed cardiac hemangioma.

**Conclusion:**

Cardiac hemangiomas can rarely grow larger than 5 cm, cause few symptoms, and are easily confused with atrial myxomas. Hepatomegaly and ascites may be signs of cardiac hemangioma.

## 1 Introduction

Cardiac hemangiomas are benign tumors with an incidence of around 3% of all cardiac tumors [1]. They are most commonly located in the right or left ventricle or in the right atrium [2]. The first report of cardiac hemangioma was published by Uskoff and colleagues in 1893 [3]. Cavernous hemangioma is the most common type [4]. Patients are mostly asymptomatic as cardiac hemangiomas are slow-growing tumors and usually do not metastasize. With an average size of 52.3 mm, they may seldom cause pericardial effusion, asymptomatic murmur, arrhythmias, hemopericardium or cardiac tamponade, dyspnea, complete heart block or even sudden death [5]. When symptoms occur, they are due to compression of cardiac structures or obstruction of outflow tracts [2]. Surgery is the treatment of choice because of the potential risk of embolism, rupture, and sudden death. Radiotherapy can be reserved for inoperable patients [6]. Other therapies include corticosteroids, β-blockers, interferon-α, anticancer drugs (such as vincristine, cyclophosphamide) [5,6].

We present a rare case of giant right atrial hemangioma in a patient symptomatic for ascites.

## 2 Case report

**Figure 1:**
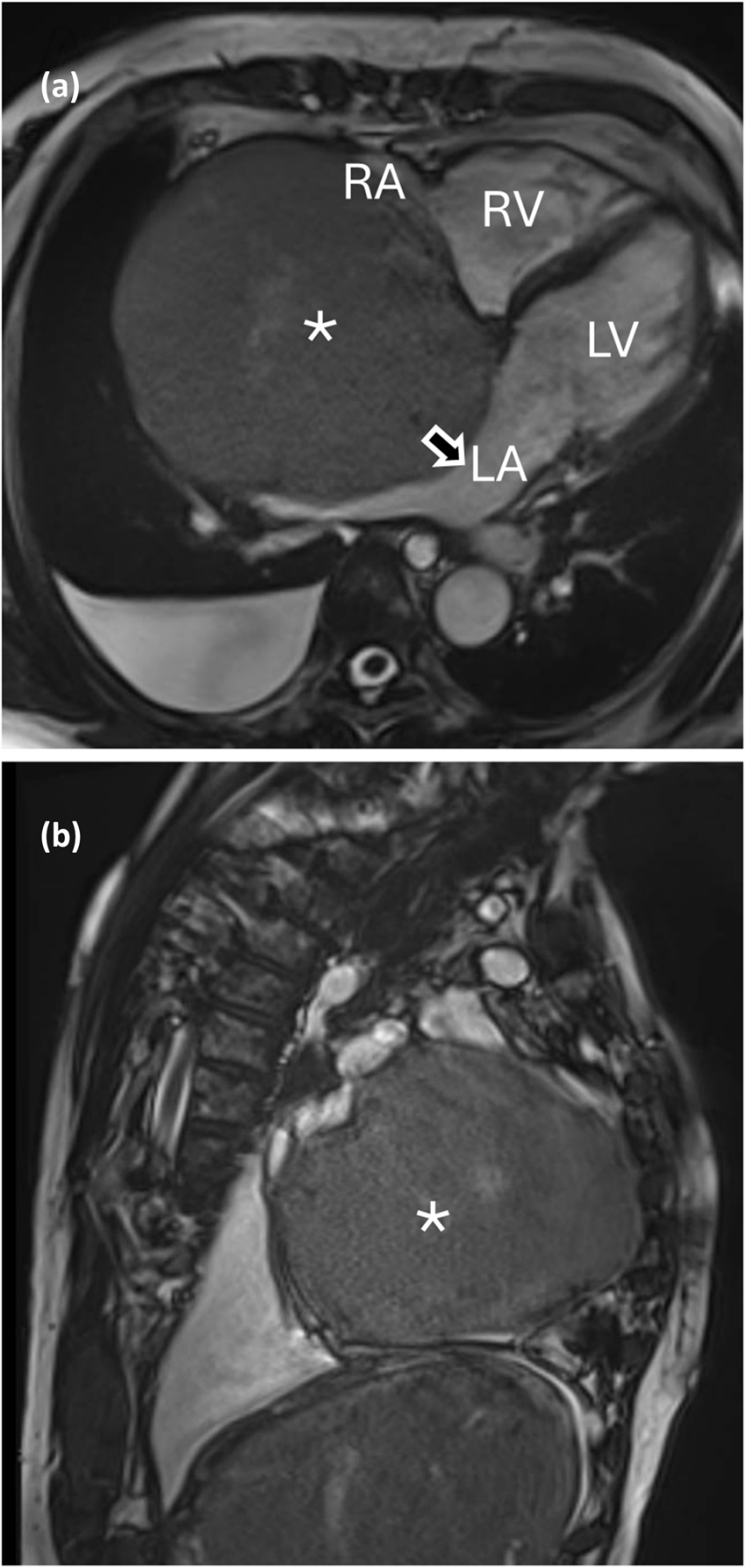
(a) and (b) Tumor in the MRI. RV: right ventricle; LV: left ventricle, LA: left atrium, RA: right atrium, arrow: interatrial septum, *: intracardiac mass.

A 72-year-old woman, active smoker with a past medical history of COPD was admitted after 6 months of abdominal swelling related to ascites, swollen ankles and mild jaundice. The patient had no history of alcoholism. Liver ultrasonography showed a cirrhotic liver pattern with nodular hepatic contour and changes in volume distribution, without signs of hepatic hemangiomas. Blood tests revealed high bilirubin levels (2 mg/dl) with predominant direct bilirubin. Gastroscopy showed esophageal varices without bleeding. She reported no pain or dyspnea. Lung examination was unrevealing. Diuretic treatment was started. The electrocardiogram showed sinus rhythm, with signs of right ventricular strain. Chest computed tomography scan and magnetic resonance imaging (MRI, Figure 1) of the chest showed pleural effusion in the right side and a mass of 130 mm in the right atrium. Echocardiography showed a voluminous mass (130 mm × 110 mm) in the right atrium attached to the interatrial septum obstructing blood venous return through the inferior vena cava which appeared dilated. Troponine I values were negative. An ultrasound-guided tranjugular biopsy of the cardiac mass was performed, which excluded malignant mesenchymal tumors, sarcomas or organized clots. Histological examination suspected atrial myxoma. A positron emission tomography scan revealed heterogeneous moderate tracer’s accumulation in correspondence to the cardiac mass.

**Figure 2:**
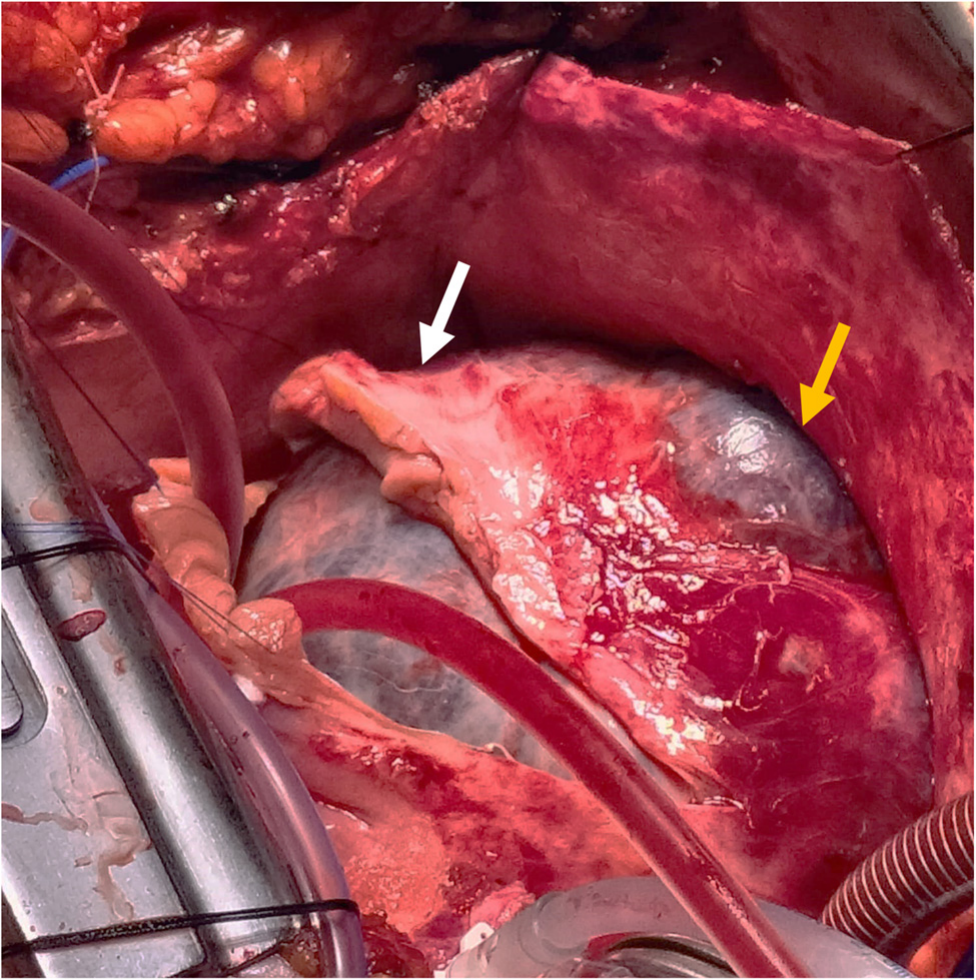
Surgical view of the cardiac tumour. White arrow: right atrial wall, yellow arrow: atrial wall eroded by the neoplasm.

**Figure 3:**
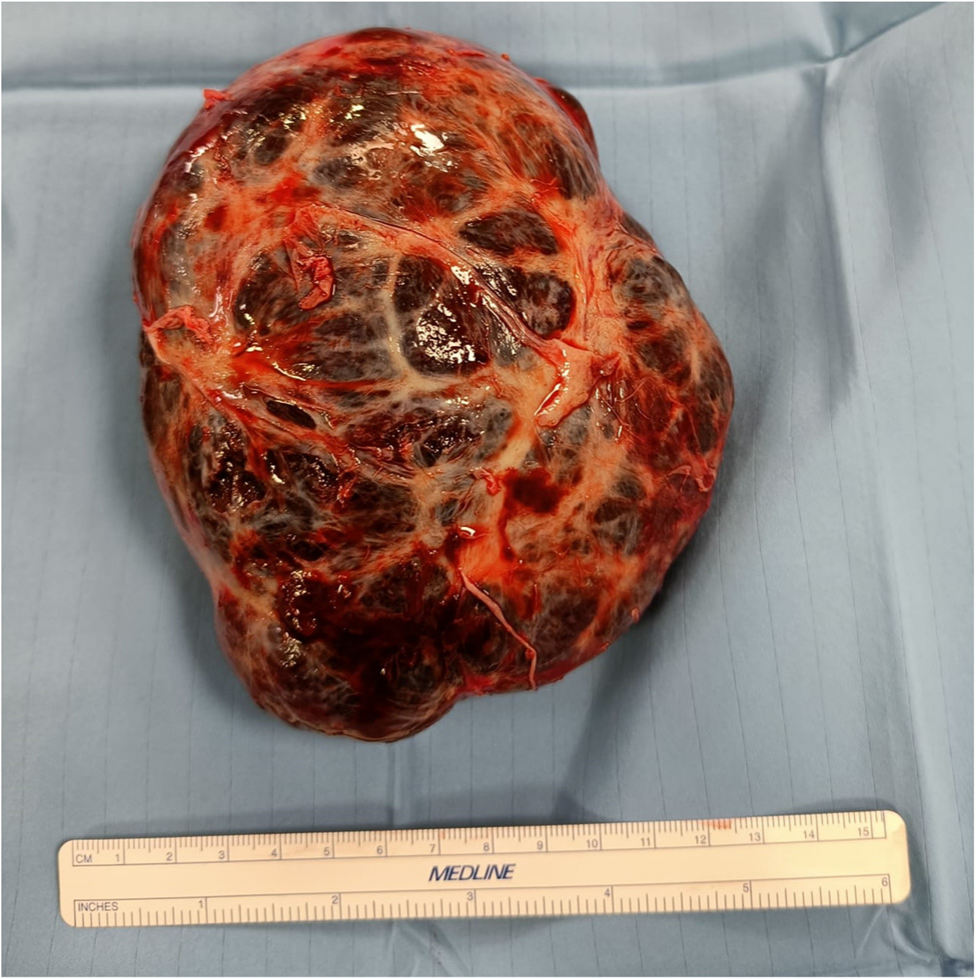
Excised mass.

**Figure 4:**
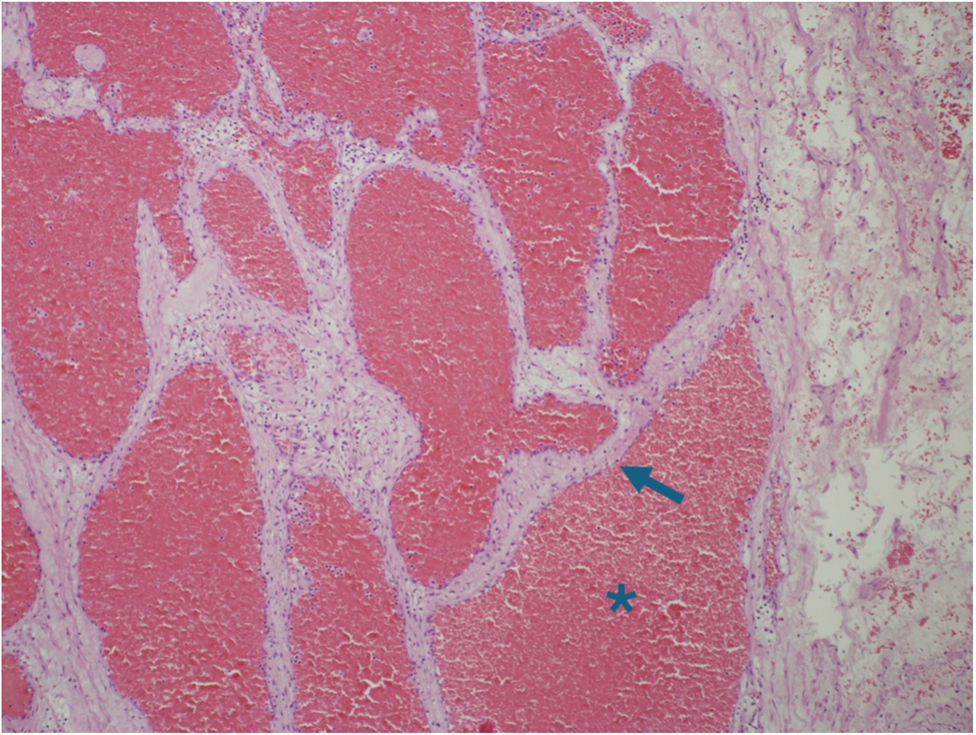
Microscope Specimen. The section shows a well-circumscribed tumor, composed of lobules of cystically dilated vascular spaces filled with blood (*) and lined by flat endothelial cells (arrow) without cytological atypia (Hematoxylin & Eosin, 100×).

The patient was scheduled for surgical excision of the cardiac mass. Radical en bloc mass resection was achieved through a right atrial approach with resection of right atrial wall, pericardium and right pleura as the tumor eroded these structures and partially replaced them (Figure 2). The tumor peduncle was located on the atrial septum, eccentric with respect to the fossa ovalis. The tricuspid valve was not affected by the disease. It was necessary to reconstruct the wall of the right atrium and the superior and inferior atriocaval junctions with bovine pericardial patch. Surgery was performed with a median sternotomy access and the use of the cardio-pulmonary bypass. Ascending aorta, superior vena cava and right femoral vein were used as arteriovenous cannulation sites. Cardiopulmonary bypass (CPB) was used and Custodiol cardioplegia was administered in the ascending aorta after clamping. CPB time was 190 min. The excised mass had a dimension of 13 cm × 7 cm × 6 cm and a weight of about one kilo. It was purplish in color, of elastic consistency with dark red spongy sections (Figure 3). The histological diagnosis was cardiac hemangioma (Figure 4).

Postoperative course was uneventful. Postoperative echocardiographic examination showed no residual mass in the right atrium, moderate tricuspid regurgitation due to chordal rupture. At 1 year follow-up the patient is alive and asymptomatic. Cancer recurrence or relapse did not occur.


**Informed consent**: Explicit informed consent and authorization for the use of the photographic image were obtained from the participating patient.

## 3 Discussion

Secondary cardiac tumors are much more common than primary ones. In autopsy series, three-quarters of primary cardiac tumors are benign, mostly myxomas, and one-quarter are malignant [1]. Cardiac hemangioma is a rare tumor that originates from abnormal hyperplasia or dilatation of small arterioles, venules and capillaries. It represents 1–2% of primary cardiac tumors which in turn have an incidence of 17 in a million on autopsy findings [1,4–6].

It occurs at all ages but it is mostly diagnosed in middle-aged patients [7,8]. The differential diagnosis includes cardiac thrombus and atrial myxoma. Hemangioma can be confused with the latter especially if a peduncle attached to the interatrial septum is present [5]. The natural history of cardiac hemangioma is unknown although it appears to be a slow-growing tumour. In most cases it is localized in the right heart and patients were usually asymptomatic. Symptoms may occur for obstruction of the cardiac chambers which impairs the blood flow [6,9,10]. Signs and symptoms include decreased exercise tolerance, syncope, angina, stroke, systemic embolism, cyanosis, attack of stuffiness, nausea and vomiting, heart murmur, systemic congestion, right ventricular outflow tract obstruction which resembles pulmonary stenosis, shortness of breath, pericardial effusion and hemopericardium, renal failure, sudden death, atrioventricular block, myocardial ischemia by direct coronary compression, coronary steal, Ebstein’s anomaly, consumptive coagulopathy [11–20]. In our case the patient presented signs such as abdominal swelling, ascites and hyperbilirubinemia, usually pathognomonic of malignant tumors of the abdomen. Due to this, the diagnosis of cardiac hemangioma was not immediately suspected, moving towards an abdominal pathology.

Hemangiomas are usually 5 cm or less in size [5]. In the present case, this 13 cm large giant cardiac tumour did not cause severe symptoms. Haemangiomas with heterogeneous shapes and behaviours are described in the literature. This kind of tumours can develop longitudinally and enter the valvular orifices mimicking a valvular pathology or enlarge and erode the surrounding cardiac structures as in this case, where a portion of the neoplasm replaced the atrial wall.

Successful conservative management has been reported with the use of corticosteroids, β-blockers, interferon-α, and anticancer drugs [5]. The cornerstone of the treatment of infants with haemangioma is the glucocorticoid therapy and only about 16% of haemangioma patients do not respond to glucocorticoid [21]. Beta-receptor blockers have more recently replaced glucocorticoids for propranolol as first-line agents to treat hemangiomas [22]. Interferon-α inhibits angiogenesis by suppressing the proliferation of haemangiomas [23].

Vincristine, cyclophosphamide, bevacizumab, and rapamycin can reduce the dose of glucocorticoids, even contribute to glucocorticoids withdrawal [24].

In adults, the treatment of choice remains surgery in presence of symptoms and diagnostic uncertainty. Surgery can be complex due to the amount of structures to be reconstructed [25]. Due to the anatomy and the high risk of sudden cardiac death, our patient underwent surgical excision. Depending on the location of the tumour, the surgical incision may vary. A median sternotomy is the standard access, minimally invasive surgery via a lateral thoracotomy may be considered. A left transatrial approach could be considered if the tumour is situated close to the the mitral valve, a right atriotomy is mandatory for the tumours of the right atrium or interatrial septum, and a ventriculotomy is necessary for the ones located in the ventricles [25,26].

## 4 Conclusions

Cardiac hemangiomas can reach considerable dimensions. Very often they are confused with atrial myxomas because of a pedicle attached to the interatrial septum. Preoperative biopsy may be inconclusive and suspect the wrong tumour. Right atrial giant hemangiomas are rare but should be considered in the differential diagnosis of ascites in patients with unknown cirrhotic disease. Surgical treatment resulted to be safe and effective.
